# The Impact of Economic Income on BMI Trajectory Groups in Chinese Elderly Individuals: A Population-Based Longitudinal Study

**DOI:** 10.3390/nu17010034

**Published:** 2024-12-26

**Authors:** Yecheng Yao, Qiya Guo, Caicui Ding, Ying Zhou, Chao Song, Yan Zhang, Weiyan Gong, Fan Yuan, Zheng Chen, Tanchun Yu, Xinyue Wu, Li He

**Affiliations:** National Institute for Nutrition and Health, Chinese Center for Disease Control and Prevention, Beijing 100050, China; yaoyc@ninh.chinacdc.cn (Y.Y.); wuxy@ninh.chinacdc.cn (X.W.)

**Keywords:** economic income, BMI trajectories, CLHLS-HF, the elderly

## Abstract

Objective: The objective of this study is to gain insights into the influence of income on the body mass index (BMI) locus in the elderly population. Methods: The Chinese Longitudinal Healthy Longevity and Happy Family Study (CLHLS-HF) was included at baseline (2008) for participants aged 65 years and older. The total number of participants analyzed in this study was 7555. A population-based trajectory model (GBTM) was used. The economic income level was an independent variable and adjusted for age, gender, ethnicity, education, marriage, and physical activity (Model 1), and the baseline BMI value was added in Model 2, with a quadratic of the income added in Model 3. A sensitivity analysis was adopted. Results: Three BMI trajectory groups were identified using GBTM and were labeled as “overweight”, “normal”, and “obesity”. After adjusting for covariates (Model 1), with the increase in economic income, the risk of the elderly transitioning into the overweight trajectory group and the obesity trajectory group was relatively increased. When the baseline BMI values were adjusted for Model 2, the effect of economic income on the overweight and obesity trajectories was enhanced. A sensitivity analysis was performed, and it was found that the result of the positive impact of economic income on the BMI trajectory group was robust. Conclusions: The higher the income, the greater the risk of Chinese elderly individuals developing the overweight or obesity trajectory. It is suggested that elderly individuals with higher economic income especially need interventions and nutrition education to help them acquire nutrition knowledge for a healthy lifestyle. The positive impact of economic income on the BMI trajectories of the elderly provides further directions for preventing and controlling obesity in the elderly.

## 1. Introduction

Over the past few decades, obesity has increased at an alarming rate in both developed and developing countries, and the number of people who are classified as overweight and obese is greater than the number of people who are classified as underweight [1,2,3,4]. As evidence suggests that overweight and obesity are closely associated with chronic diseases, such as adverse metabolic effects on blood pressure, cholesterol, diabetes, and certain types of cancer [5,6,7,8,9,10,11], the increasing prevalence of overweight and obesity has become one of the greatest global public health concerns worldwide [12,13,14].

This trend not only poses a serious threat to individual health, but also increases the health burden, and thus, the problem of overweight and obesity has become a major challenge and a serious hidden danger in the global public health field [15,16,17]. In Canada, the fiscal burden of obesity was estimated to be CAD 22,974 million in 2021 [18]. The economic burden of obesity across Saudi Arabia was estimated to be USD 6.4 billion for treatment and management [19]. In addition, with a 15% lower body weight at baseline, USD 221 million in cumulative savings was estimated, corresponding to USD 2205 in savings over 5 years in the United States [20].

In China, with faster economic growth and urbanization, the prevalence of overweight and obesity is rising rapidly and continues to grow [21]. The current health burden of overweight and obesity in China has increased and will increase in coming years. The projected number of adults with the prevalence of overweight and obesity is estimated to be 810.65 million in 2030 [22]. In addition, according to the National Bureau of Statistics, China’s increasing elderly population is both a challenge and an opportunity, and it is reported that the population of Chinese people on the mainland aged 60 or above has reached 264.02 million, including 190.64 million people aged 65 or above, or 13.5 percent of the total population in 2021 [23].

The body mass index (BMI) is measured to determine overweight and obesity, and increased BMI has become a major public health issue worldwide and attracted the attention of many scholars [24,25,26,27]. Additionally, economic income (as the indicator of socioeconomic status, SES) was a key determinant studied. One cross-sectional study found that children with lower family incomes tend to have higher risk of obesity than children with higher family incomes in California [28]. In addition, a review of 191 studies found that the prevalence of obesity in individuals with lower SES is higher than that for participants with higher SES [29].

These studies, however, were based on BMI at one point in time to assess the effect of economic income on overweight/obesity risk and were unable to assess the effect of economic income on the long-term change in BMI. To avoid this limitation, BMI trajectories [30] were introduced in this study to make better use of data derived from the Chinese Longitudinal Healthy Longevity and Happy Family Study (CLHLS-HF).

Therefore, this study aimed to explore the impact of economic income on the BMI trajectory group of Chinese elderly individuals and to provide a basis for formulating corresponding effective obesity prevention and control strategies.

## 2. Materials and Methods

### 2.1. Study Design and Participants

The data for this study were obtained from the CLHLS-HF [31], a long-term longitudinal study conducted among community residents aged 65 and older in China. CLHLS-HF was launched in 1998 and conducted every 2–3 years, and it was jointly conducted by the Centre for Healthy Aging and Development Studies at Peking University and the Chinese Centre for Disease Control and Prevention.

The most recent (eighth round) data were updated in 2018. As the initial four rounds of data lacked the requisite height data to calculate BMI, data from the fifth round (in 2008) to the eighth round (in 2018) were utilized in this study. Participants aged 65 years and older at baseline (in 2008) were included, and then we further limited the participants by selecting only those who had undergone at least two assessments of height and weight. Those who did not undergo both height and weight assessments in the same round were excluded to avoid not being able to obtain BMI for a cohort-based trajectory analysis. In the end, a total of 7555 participants were included in the study.

### 2.2. BMI Trajectory Recognition

BMI measurements and standardized examination procedures were used to measure the degree of overweight/obesity and the health of participants in successive surveys. BMI is calculated by dividing the weight (in kilograms) of the corresponding survey wave by the square of the corresponding height (in meters) [32].

The formula is as follows:

Body Mass Index (BMI) = weight (kg)/height^2^ (m^2^)

In order to avoid the impact of extreme BMI outliers on the study, data exceeding a 99% BMI value were identified as missing values in this study.

The GBTM (Group-Based Trajectory Model) [33] is designed to identify groups of individual trajectories that behave similarly in this study. Based on the latent class model, the GBTM attempts to divide the observed individual trajectories into several potential groups, each with a similar pattern of trajectories. This approach allows researchers to uncover potential group structures hidden in the data without knowing the group membership beforehand. The optimal trajectory model is selected by the minimum Bayesian Information Criterion (BIC), minimum Akaike Information Criterion (AIC), minimum logarithmic likelihood (ll) and maximum entropy. In addition, the average posterior probability (AvePP, >0.7) and the group member prediction probability (PPGM, >5%) are used as additional statistical indicators. The clinical significance of the locus group was also considered in this model selection process.

### 2.3. Independent Variable and Covariates

SES includes economic income, economy sufficiency, and economic status. Economic income refers to the total income of the participants in the past year (yuan); economy sufficiency refers to whether the participants are satisfied with all sources of income; and economic status refers to the participants’ level of well-being compared to their local area, assessed on a scale of 1–5, with higher scores indicating greater financial hardship.

To adjust the model to accommodate background characteristics and to obtain a complete explanation of the effect of economic income on BMI trajectories, covariates were selected, including demographic characteristics and physical activity status.

Demographic characteristics include age (grouped by age 65–69, 70–79, and 80 years and older), gender (female or male), place of residence (rural or urban), nationality (Han or other ethnic groups), education (illiterate or non-illiterate), marital status (married or separated/divorced/widowed/unmarried).

Physical activity status includes physical activity, physical labor, and leisure activities. Whether physical activity is or has been performed regularly (purposeful fitness activities) is considered. Physical labor conditions refer to whether the participants have often engaged in physical labor in the past. The number of leisure activities refers to the types of leisure activities participated in every day, including housework, gardening and pet raising, reading books and newspapers, raising poultry and livestock, playing cards or mahjong, watching TV and listening to the radio, and participating in organized social activities. According to the types of leisure activities participated in every day, the participants are divided into no leisure activities, those who participate in one leisure activity a day, and those who participate in at least two leisure activities a day.

In order to avoid the influence of missing values on model estimation, the missing covariate data are interpolated by the missForest algorithm based on a random missing hypothesis.

### 2.4. Statistical Analysis

The statistical analysis was conducted using R software (version 4.2.1) and Stata software (version 13). Continuous variables were reported as mean ± standard deviation and compared using the Mann–Whitney or Kruskal–Wallis tests. In contrast, the categorical variables were described statistically by *n* (%) and compared using the Chi-square test or Fisher exact probability method. Multicategorical Logistic regression was used to assess the association between economic income and BMI trajectory groups.

Three multi-classification Logistic regression models (nnet packages) were designed to assess the effect of income levels on BMI trajectory groups. First, income level was included as an independent variable, adjusted for age, sex, ethnicity, education, marriage, and physical activity (Model 1). Secondly, in order to exclude the positive correlation of the baseline BMI to the BMI trajectory group, the baseline BMI value was also included on the basis of Model 1. Finally, previous studies have found the influence of quadratic income on the BMI value [34], so quadratic income was added on the basis of Model 2. The results are reported as OR, 95% CI, and *p*-value.

Sensitivity analyses were conducted to test the robustness of the results. Since a trajectory analysis is more stable for participants with more observations, 4548 individuals who completed at least three rounds of BMI assessment were included for group-based trajectory identification and model analysis.

## 3. Results

### 3.1. Baseline Characteristics

#### 3.1.1. Grouping of BMI Trajectories

**Figure 1:**
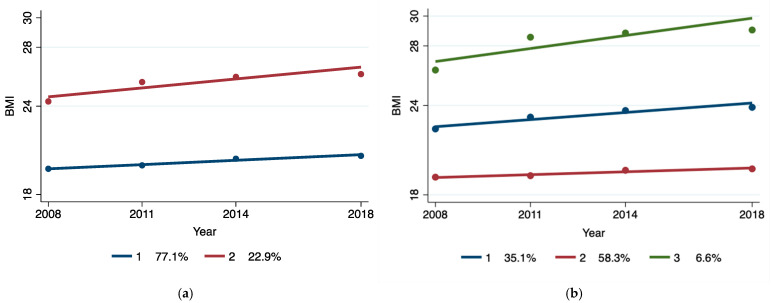
Group identification of BMI trajectories in elderly individuals completing at least two rounds of BMI. (**a**) For 2 trajectories; (**b**) for 3 trajectories.

Three groups of BMI trajectories were identified using the GBTM. As shown in Table 1 and Figure 1, based on the BIC, AIC, ll values, Entropy, and average posterior probabilities, as well as the sample sizes for each locus, the three groups of BMI trajectories were the most realistic. Most participants (58.3%) remained relatively stable during the follow-up; some participants (35.1%) progressed from a normal BMI at baseline (BMI < 24) to overweight in 2018 (BMI > 24). The BMI trajectory of 6.6% of participants quickly jumped from overweight (BMI > 24) at baseline to obesity (BMI > 28). Therefore, the three BMI trajectory groups were labeled as “overweight”, “normal”, and “obesity”.

#### 3.1.2. Baseline Characteristics of Participants

A total of 7555 participants were included in the analysis. The distribution of baseline characteristics of different BMI trajectory groups (Table 2) was compared, and significant differences in the baseline BMI values were observed among the different BMI trajectory groups (*p* < 0.001). Of the participants with a normal baseline BMI, 70.9% (*n* = 4465) remained in the normal trajectory group, while 27.4% (*n* = 1726) transitioned into the overweight trajectory group. Of the participants who were overweight at baseline, 72.4% (*n* = 744) were in the overweight trajectory group, and 18.2% (*n* = 187) were in the obesity trajectory group. The results show that those aged 80 years and older tended to be in the normal trajectory group (70.4%), while the 65–69-year-old age group tended to be in the overweight trajectory group (49.9%). Compared with females, there were more males in the overweight trajectory group, while there were fewer males in the obesity trajectory group. Compared with the corresponding reference groups, the overweight and obesity trajectory groups significantly accounted for more participants in the urban, Han, non-illiterate, and married groups (*p* < 0.001). In addition, the proportion of individuals in the overweight and obesity trajectory group presently participating in physical activity was relatively higher, while the proportion of individuals in the overweight and obesity trajectory group participating in physical labor was relatively lower (*p* < 0.001). Participants who participated in one or at least two leisure activities per day had higher rates of overweight and obesity (*p* < 0.001).

The comparison of economic conditions shows that the economic income of different BMI trajectory groups was significantly different (*p* < 0.001).

### 3.2. Association of Economic Income with BMI Trajectory Groups

As shown in Table 3, after adjusting for covariates (Model 1), with the increase in economic income, the risk of the elderly transitioning into the overweight trajectory group and the obesity trajectory group was relatively increased, respectively. In addition, compared with the normal trajectory group, participants who were economy sufficient, had a higher number of leisure activities, presently participated in physical activity, had an urban place of residence, were of Han nationality, and married were at a higher risk for being in the overweight trajectory group, while participants with more physical labor and in a higher age group were at a lower risk for being in the overweight trajectory group. Participants who were presently participating in physical activity and had an urban place of residence had a higher risk of being in the obesity trajectory group, while those who were in a higher age group and male had a lower risk of being in the obesity trajectory group.

When the baseline BMI values were adjusted for the model (Model 2), the effect of economic income on the overweight and obesity trajectories was enhanced. Participants with higher economic status scores (indicating greater financial hardship) and in higher age groups had a lower risk of being in the overweight trajectory group. In the analysis of the obesity trajectory group, it was found that the risk of being in the obesity trajectory group was higher in individuals in an urban residence, and the risk was lower in higher age groups and in the Han nationality group. After that, the square of economic income was incorporated into the model (Model 3), and it was found that the square of economic income had no significant influence on the overweight trajectory group and the obesity trajectory group.

### 3.3. Sensitivity Analysis

**Figure 2:**
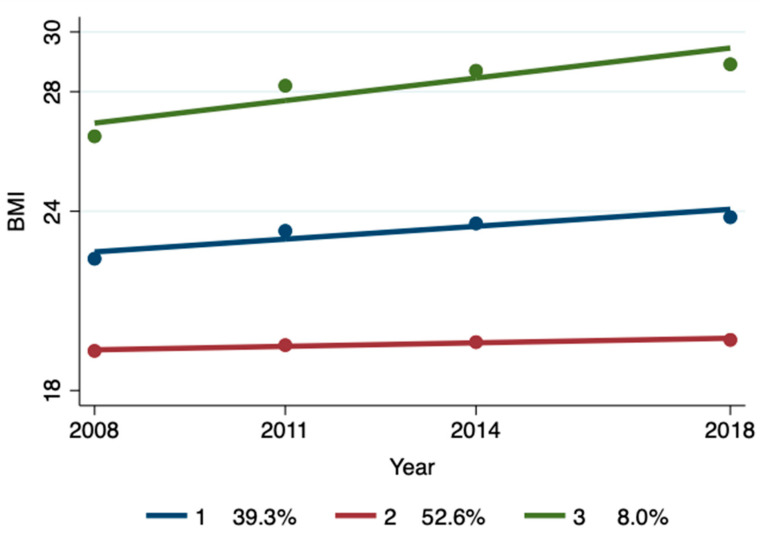
BMI trajectory group identification for completing at least three rounds of BMI assessment.

**Figure 3:**
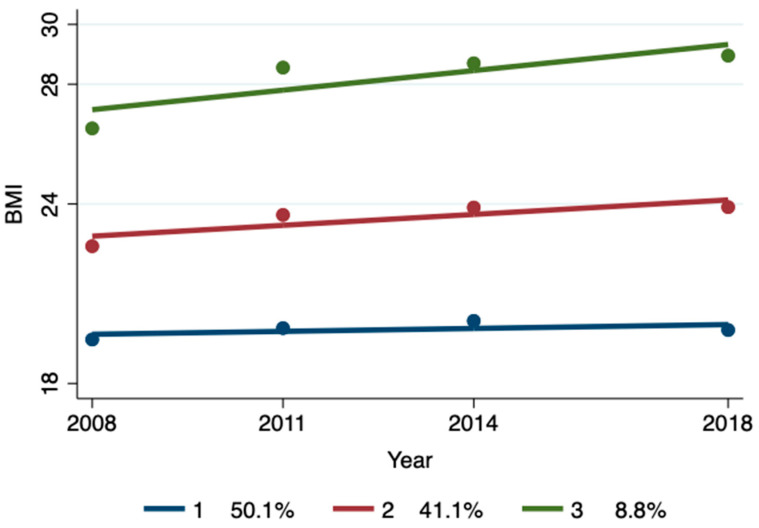
BMI trajectory group identification for completing four rounds of BMI assessment.

A sensitivity analysis was performed on 4548 samples that completed at least three rounds of BMI assessment and 2001 samples that completed four rounds of BMI assessment (Figure 2 and Figure 3; Table 4 and Table 5), and it was found that the group trajectory and multiple Logistic regression results remained basically unchanged, indicating that the conclusion of the positive impact of economic income on the BMI trajectory group was robust.

## 4. Discussion

### 4.1. Findings and Potential Interpretations

This study found a positive effect of economic income on the occurrence of overweight and obesity trajectories in elderly people. In addition, it found that the elderly individuals in the older age group had a relatively lower risk of developing overweight and obesity trajectories. In addition, with economic growth and urbanization, the elderly individuals living in urban areas with higher economic income have a relatively higher risk of developing obesity trajectories; this may be due to a lack of education, unhealthy dietary habits changing from low-density food to high-density food, as well as increased sedentary time, which contribute to obesity [35,36,37,38,39].

This trend is consistent with previous studies showing that the BMI with higher economic income may be greater than that with lower economic income. It was reported that having overweight or obesity was more likely among those in high SES groups by analyzing four rounds of India’s National Family Health Surveys in a study population consisting of 1,244,149 women and 227,585 men in India [40]. There was a positive change in overweight/obesity, which was found as the proportion of women who attended secondary school and higher levels of education increased, and the possible explanation is that these women may have higher incomes and might be more exposed to high-density food in Ethiopia [41].

There are many reasons why there is a positive effect of economic income on overweight and obesity; it may be associated with different dietary habits that are triggered by unhealthy lifestyles, including ultra-processed foods, neighborhood built environments, as well as increased sedentary behaviors [42,43,44,45,46,47]. In recent decades, economic income has increased, and the food system has changed from homemade traditional food to purchased food, such as ultra-processed food, which is characterized by being dense in energy and poor in nutrients [48,49]. Ultra-processed food, as the away-from-home sector, is complex and is composed of both global and national chains and small vendors, allowing for its high availability and accessibility [50,51,52]. Elderly individuals tend to dine out more frequently and increase their ultra-processed food consumption. However, evidence demonstrates that greater ultra-processed food consumption has a positive impact on weight gain and overweight/obesity [53]. In a large French cohort with 110,260 adult participants, it was reported that a higher consumption of ultra-processed food is associated with an increase in BMI and higher risks of overweight and obesity [54]. In the UK National Diet and Nutrition Survey with 6143 participants, it was found that a 10% increase in the consumption of ultra-processed foods is associated with an 18% increase in the prevalence of obesity in men and a 17% increase in women [55]. As for older adults in the Seniors Study on Nutrition and Cardiovascular Risk in Spain, it was found that higher ultra-processed food consumption is positively associated with incident obesity in older adults in Spain [56]. There is a need to change the obesogenic environment to support individuals to reduce their ultra-processed food consumption [50,57].

In addition, the accumulation of nutrition knowledge in the elderly lags behind the improvement in economic level, that is, the knowledge and awareness of nutrition and health have not been improved correspondingly, as the major objective of overweight and obesity prevention is to prevent and control related chronic diseases at the source and focus on maintaining the elderly individuals’ health rather than merely treating illnesses [36,58].

### 4.2. Implications and Suggestions

As economic income is still one of the important factors affecting the health and nutrition of the elderly in China’s transitional economy, elderly individuals in China with greater economic income have increased nutrition-related conditions, such as overweight and obesity, caused by a lack of education, unhealthy dietary habits, and increased sedentary time.

To tackle the increasing overweight or obesity rates, China has introduced a series of initiatives aimed at raising public awareness of weight management. Chinese authorities, including the National Health Commission (NHC) and the ministries of education and civil affairs, have issued the document to launch a three-year campaign to enhance the public’s weight management since June 2024 [59]. It is noted that the abnormal weight status of elderly groups of people should be improved over time.

China has made many efforts to tackle obesity; however, current efforts to tackle overweight and obesity are gravely inadequate [60,61]. In order to effectively control BMI and reduce the prevalence of overweight or obesity, the following suggestions are put forward: Firstly, the authorities should carry out a series of measures on reasonable diet and nutrition-related education, as well as provide a supportive environment for the whole society, especially for the elderly, so that the elderly can obtain more health knowledge, form good diet habits, and construct healthier diets so as to reduce the occurrence of overweight or obesity. Secondly, it is suggested that elderly individuals who live in urban areas with higher income may need more nutrition education to help them acquire nutrition knowledge and form a healthy lifestyle so as to improve their abnormal weight status and reduce overweight or obesity. Moreover, the community should be encouraged by providing a series of practical health education sessions for elderly individuals with an overweight or obesity risk by making full use of various lectures, posters, and other media according to the Dietary Guidelines for Chinese Residents (2022) [62] to improve their awareness of weight management. Thirdly, considering elderly individuals with malnutrition, the authorities should appropriately increase subsidies to these low-income individuals so that economic income can play a role for the elderly with an underweight status associated with food insecurity, helping them return to a normal weight level.

### 4.3. Limitations and Strengths

There are still potential limitations in this study, as it is limited due to the public data not being updated to the latest data, so this study’s conclusions on BMI trajectories cannot be tracked to the latest data. It would be beneficial to include more recent income data or adjust for potential income fluctuations over the study period. In addition, the self-reported data from the CLHLS-HF study may introduce bias, and sensitivity analyses were conducted in the study to increase the robustness and reliability of the results. Moreover, only four rounds of CLHLS-HF were analyzed in this study as the initial four rounds of data lacked the requisite height data to calculate the BMI, but the effect on the obesity trajectory group was still significant.

Although the prior literature has found an effect of economic income on overweight and obesity or BMI, since it was based on the BMI at one point in time, it was unable to assess the effect of economic income on the long-term change in BMI, especially among the elderly. This article has studied the impact of economic income on BMI trajectories, filling in the blanks of previous studies in this area among the elderly population, and a sensitivity analysis was conducted to make the results more scientific and reliable, which will not only improve the health of the elderly, but also encourage policy makers to improve public health.

### 4.4. Directions for Future Studies

Given the pressing necessity to control and prevent overweight or obesity, directions for future studies derived from our findings still need exploring, and it would be fruitful to investigate the pathways that link economic income with BMI, that is, the mechanisms by which economic income affects nutrition-related conditions like overweight or obesity, such as dietary factors, access to healthcare, diet quality, and social support. And other factors with an influence on socioeconomic status, like wealth, job stability, social support, pension status, and health costs, could also be explored in future studies, which could provide more comprehensive insights into the ways in which socioeconomic status influences obesity and other health outcomes in the elderly. Moreover, future studies could investigate similar questions in other countries with different economic structures, healthcare systems, and cultural attitudes toward aging, income, and obesity to enhance the generalizability of the findings. These future studies may require multidisciplinary knowledge, stimulating effective behavior change, and establishing appropriate environments. Understanding these mechanisms or interventions, as well as conducting further investigations of the elderly, will aid in decision making and policy implementation to tackle obesity.

## 5. Conclusions

This study found a positive effect of economic income on the BMI trajectories of the elderly. The higher the economic income, the greater the risk of developing an overweight or obesity trajectory. This study provides further insights into preventing and controlling obesity in elderly people.

## Figures and Tables

**Table 1:** Identification of BMI trajectory groups.

BMI Trajectory	Orders	BIC	AIC	ll	Entropy	AvePP_G1	AvePP_G2	AvePP_G3	AvePP_G4	PPGM1 (%)	PPGM2 (%)	PPGM3 (%)	PPGM4 (%)
One	I	−59,306.79	−59,296.40	−59,293.40									
Two	II	−57,216.24	−57,195.45	−57,189.45	0.760	0.945	0.877			77.084	22.914		
Three	III	−56,715.03	−56,683.85	−56,674.85	0.697	0.808	0.882	0.862		35.083	58.342	6.575	
Four	IIII	−56,613.72	−56,572.14	−56,560.14	0.66	0.847	0.78	0.736	0.832	1.959	11.966	38.24	47.835

**Table 2:** Comparison of baseline features of different BMI trajectory groups.

Variable	Normal Trajectory Group (N = 4565)	Overweight Trajectory Group (N = 2535)	Obesity Trajectory Group (N = 455)	*p*
BMI	19.1 ± 2.3	22.8 ± 2.7	26.7 ± 4.0	<0.001
BMI Classification				<0.001
normal	4465 (70.9)	1726 (27.4)	103 (1.6)	
overweight	96 (9.3)	744 (72.4)	187 (18.2)	
obesity	4 (1.7)	65 (27.8)	165 (70.5)	
income	1.4 ± 1.4	1.6 ± 1.5	1.7 ± 1.6	<0.001
Economy				<0.001
insufficient	1112 (65.8)	499 (29.5)	79 (4.7)	
sufficient	3453 (58.9)	2036 (34.7)	376 (6.4)	
economic status	3.1 ± 0.6	3.0 ± 0.7	2.9 ± 0.6	<0.001
Age				<0.001
65–69	429 (39.2)	546 (49.9)	120 (11.0)	
70–79	1077 (50.9)	863 (40.8)	175 (8.3)	
≥80	3059 (70.4)	1126 (25.9)	160 (3.7)	
Gender				<0.001
female	2581 (63.5)	1219 (30.0)	264 (6.5)	
male	1984 (56.8)	1316 (37.7)	191 (5.5)	
Residence				<0.001
rural	2991 (64.1)	1448 (31.0)	230 (4.9)	
urban	1574 (54.5)	1087 (37.7)	225 (7.8)	
Nationality				<0.001
other	325 (69.1)	123 (26.2)	22 (4.7)	
Han	4240 (59.8)	2412 (34.0)	433 (6.1)	
Education				<0.001
illiterate	2810 (66.5)	1202 (28.4)	216 (5.1)	
non-illiterate	1755 (52.8)	1333 (40.1)	239 (7.2)	
Marital Status				<0.001
other	2927 (67.6)	1193 (27.5)	213 (4.9)	
married	1638 (50.8)	1342 (41.7)	242 (7.5)	
Physical Activity				<0.001
none	2716 (64.5)	1291 (30.7)	202 (4.8)	
previous	489 (63.7)	234 (30.5)	45 (5.9)	
present	1360 (52.8)	1010 (39.2)	208 (8.1)	
Physical Labor				<0.001
does not participate	625 (53.6)	455 (39.1)	85 (7.3)	
participates	3940 (61.7)	2080 (32.6)	370 (5.8)	
Number of Leisure Activities				<0.001
0	1050 (72.1)	350 (24.0)	56 (3.8)	
1	1360 (63.9)	655 (30.8)	114 (5.4)	
≥2	2155 (54.3)	1530 (38.5)	285 (7.2)	

**Table 3:** Effect of economic income on BMI trajectory group.

Variable	Model 1		Model 2		Model 3	
	OR (95%CI)	*p*	OR (95%CI)	*p*	OR (95%CI)	*p*
Overweight trajectory group						
Income	1.06 (1.02, 1.10)	0.004	1.07 (1.02, 1.12)	0.006	1.10 (0.98, 1.24)	0.095
Income^2^	-	-	-	-	0.99 (0.98, 1.01)	0.559
Economy sufficient	1.21 (1.06, 1.40)	0.007	1.13 (0.96, 1.34)	0.153	1.13 (0.95, 1.34)	0.163
Economic status	1.07 (0.98, 1.17)	0.153	1.19 (1.06, 1.34)	0.002	1.20 (1.07, 1.34)	0.002
Number of leisure activities	1.13 (1.05, 1.21)	0.001	1.04 (0.95, 1.13)	0.409	1.04 (0.95, 1.13)	0.397
Physical activity (ref: none)						
Previous	0.96 (0.8, 1.15)	0.655	1.06 (0.86, 1.32)	0.571	1.06 (0.86, 1.32)	0.571
Present	1.21 (1.08, 1.36)	0.001	1.08 (0.94, 1.25)	0.268	1.08 (0.94, 1.25)	0.276
Physical labor	0.84 (0.72, 0.97)	0.017	0.88 (0.73, 1.05)	0.145	0.88 (0.73, 1.05)	0.153
Age (ref: 65–69)						
70–79	0.67 (0.57, 0.79)	<0.001	0.66 (0.54, 0.80)	<0.001	0.66 (0.55, 0.8)	<0.001
≥80	0.37 (0.31, 0.43)	<0.001	0.43 (0.35, 0.52)	<0.001	0.43 (0.35, 0.52)	<0.001
Gender (male)	1.11 (0.99, 1.25)	0.083	1.00 (0.86, 1.15)	0.979	1.00 (0.87, 1.15)	0.989
Residence (urban)	1.24 (1.11, 1.39)	<0.001	1.12 (0.97, 1.28)	0.112	1.12 (0.97, 1.28)	0.121
Nationality (Han)	1.38 (1.10, 1.72)	0.005	0.87 (0.66, 1.13)	0.293	0.87 (0.66, 1.14)	0.303
Education (non-illiterate)	1.11 (0.98, 1.26)	0.088	0.98 (0.85, 1.14)	0.837	0.98 (0.85, 1.14)	0.816
Marital status (married)	1.29 (1.15, 1.45)	<0.001	1.14 (0.99, 1.32)	0.063	1.14 (0.99, 1.32)	0.062
BMI at baseline	-	-	1.77 (1.72, 1.82)	<0.001	1.77 (1.72, 1.82)	<0.001
Obesity trajectory group						
Income	1.09 (1.02, 1.17)	0.017	1.10 (1.01, 1.21)	0.031	1.37 (1.09, 1.73)	0.008
Income^2^	-	-	-	-	0.97 (0.93, 1.00)	0.053
Economy sufficient	1.19 (0.90, 1.58)	0.227	1.09 (0.76, 1.56)	0.653	1.05 (0.73, 1.52)	0.783
Economic status	0.87 (0.72, 1.04)	0.120	0.94 (0.75, 1.17)	0.573	0.96 (0.76, 1.20)	0.704
Number of leisure activities	1.05 (0.91, 1.23)	0.489	0.93 (0.77, 1.12)	0.423	0.93 (0.77, 1.12)	0.456
Physical activity (ref: none)						
Previous	1.16 (0.82, 1.65)	0.403	1.45 (0.94, 2.24)	0.092	1.43 (0.93, 2.21)	0.105
Present	1.52 (1.22, 1.91)	<0.001	1.08 (0.82, 1.44)	0.579	1.06 (0.80, 1.41)	0.678
Physical labor	0.90 (0.68, 1.18)	0.436	1.09 (0.77, 1.54)	0.624	1.12 (0.79, 1.58)	0.538
Age (ref: 65–69)						
70–79	0.60 (0.46, 0.78)	<0.001	0.61 (0.44, 0.86)	0.005	0.63 (0.45, 0.89)	0.009
≥80	0.21 (0.16, 0.29)	<0.001	0.28 (0.19, 0.41)	<0.001	0.29 (0.19, 0.42)	<0.001
Gender (male)	0.71 (0.57, 0.90)	0.004	0.77 (0.58, 1.03)	0.075	0.78 (0.58, 1.04)	0.089
Residence (urban)	1.47 (1.18, 1.83)	<0.001	1.40 (1.06, 1.86)	0.018	1.35 (1.02, 1.80)	0.035
Nationality (Han)	1.32 (0.84, 2.08)	0.228	0.55 (0.31, 0.97)	0.038	0.57 (0.32, 0.99)	0.047
Education (non-illiterate)	1.09 (0.86, 1.38)	0.483	0.96 (0.71, 1.29)	0.768	0.93 (0.69, 1.26)	0.653
Marital status (married)	1.23 (0.98, 1.54)	0.077	0.96 (0.72, 1.27)	0.774	0.95 (0.72, 1.27)	0.737
BMI at baseline	-	-	2.67 (2.54, 2.80)	<0.001	2.67 (2.54, 2.81)	<0.001

**Table 4:** Effect of economic income on BMI trajectory group that completed at least 3 rounds of assessment.

Variable	Overweight Trajectory Group		Obesity Trajectory Group	
	OR (95%CI)	*p*	OR (95%CI)	*p*
Income	1.09 (1.03, 1.16)	0.004	1.15 (1.03, 1.28)	0.013
Economy sufficient	1.02 (0.83, 1.25)	0.861	1.01 (0.66, 1.55)	0.947
Economic status	1.15 (1.00, 1.33)	0.049	0.93 (0.71, 1.21)	0.579
Number of leisure activities	1.06 (0.95, 1.19)	0.280	0.89 (0.7, 1.12)	0.329
Physical activity (ref: none)				
Previous	1.01 (0.77, 1.34)	0.926	1.49 (0.88, 2.53)	0.134
Present	1.07 (0.90, 1.27)	0.447	1.04 (0.75, 1.46)	0.799
Physical labor	0.79 (0.63, 0.99)	0.042	0.97 (0.64, 1.47)	0.883
Age (ref: 65–69)				
70–79	0.65 (0.52, 0.80)	<0.001	0.61 (0.42, 0.88)	0.009
≥80	0.51 (0.40, 0.63)	<0.001	0.32 (0.21, 0.50)	<0.001
Gender (male)	0.92 (0.78, 1.10)	0.375	0.66 (0.47, 0.92)	0.016
Residence (urban)	1.16 (0.98, 1.38)	0.076	1.44 (1.03, 2.00)	0.031
Nationality (Han)	0.81 (0.59, 1.12)	0.211	0.50 (0.25, 0.97)	0.040
Education (non-illiterate)	0.99 (0.83, 1.18)	0.891	0.86 (0.60, 1.22)	0.386
Marital status (married)	1.09 (0.92, 1.29)	0.301	0.91 (0.66, 1.26)	0.558
BMI at baseline	1.67 (1.62, 1.73)	<0.001	2.56 (2.42, 2.72)	<0.001

**Table 5:** Effect of economic income on BMI trajectory group that completed 4 rounds of assessment.

Variable	Overweight Trajectory Group		Obesity Trajectory Group	
	OR (95%CI)	*p*	OR (95%CI)	*p*
Income	1.06 (0.96, 1.16)	0.245	1.19 (1.01, 1.39)	0.039
Economy sufficient	0.94 (0.69, 1.28)	0.699	0.92 (0.49, 1.72)	0.798
Economic status	1.09 (0.87, 1.36)	0.451	0.70 (0.47, 1.05)	0.087
Number of leisure activities	1.10 (0.91, 1.33)	0.309	0.8 (0.55, 1.16)	0.235
Physical activity (ref: none)				
Previous	1.11 (0.74, 1.68)	0.610	1.65 (0.74, 3.66)	0.218
Present	1.06 (0.82, 1.39)	0.641	1.16 (0.71, 1.89)	0.558
Physical labor	0.61 (0.43, 0.87)	0.006	0.84 (0.43, 1.62)	0.596
Age (ref: 65–69)				
70–79	0.57 (0.43, 0.75)	<0.001	0.49 (0.30, 0.81)	0.005
≥80	0.53 (0.38, 0.74)	<0.001	0.29 (0.15, 0.56)	<0.001
Gender (male)	0.85 (0.66, 1.10)	0.212	0.67 (0.41, 1.09)	0.109
Residence (urban)	1.36 (1.04, 1.76)	0.022	1.59 (0.97, 2.59)	0.064
Nationality (Han)	0.73 (0.46, 1.17)	0.192	0.34 (0.14, 0.82)	0.017
Education (non-illiterate)	1.03 (0.80, 1.33)	0.822	0.64 (0.39, 1.06)	0.084
Marital status (married)	1.27 (0.99, 1.64)	0.058	0.79 (0.49, 1.26)	0.320
BMI at baseline	1.65 (1.57, 1.74)	<0.001	2.60 (2.38, 2.85)	<0.001

## Data Availability

The Chinese Longitudinal Healthy Longevity and Happy Family Study (CLHLS-HF) dataset used in this study was publicly available at Peking University (https://opendata.pku.edu.cn/dataverse/CHADS, (accessed on 23 December 2024)).
